# Draft Genome Sequence of a Tepidicella baoligensis Strain Isolated from an Oil Reservoir

**DOI:** 10.1128/MRA.00796-20

**Published:** 2020-09-24

**Authors:** Shan Hong, Guan Wang, Jiliang Yu, Jing You, Yanfen Xue

**Affiliations:** a State Key Laboratory of Microbial Resources, Institute of Microbiology, CAS, Beijing, China; b Engineering Technology Research Institute of Huabei Oilfield Company, Renqiu, China; Georgia Institute of Technology

## Abstract

We report the draft genome sequence of Tepidicella baoligensis strain B18-50^T^, isolated from a high-temperature oil well in Baolige Oilfield, China. The estimated genome is 2.87 Mb, with 2,653 protein-coding sequences.

## ANNOUNCEMENT

*Tepidicella* species are slightly thermophilic Gram-negative rods that are distributed in geothermal areas. These organisms do not assimilate carbohydrates or polyols but use organic acids as carbon and energy sources and oxidize thiosulfate to sulfate in the presence of an assimilable carbon source (1, 2). Tepidicella baoligensis strain B18-50^T^ was isolated from the production liquid of a high-temperature oilfield in China (2). The whole-genome sequence will help in understanding its ecological function in the oil reservoir environment.

Strain B18-50^T^ was isolated from oil well production liquid from Baolige Oilfield (44°86′N, 115°81′E) in China. The liquid sample was diluted with sterilized enrichment medium (EN), plated onto EN agar, and incubated at 50°C (2). A pure strain was obtained by repeated streaking on the same medium. Strain B18-50^T^ was routinely cultured at 45°C in modified Degryse 162 medium (3) supplemented with 10 g liter^−1^ succinate, 1.0 g liter^−1^ yeast extract (Oxoid), and 1.0 g liter^−1^ tryptone (Oxoid). Genomic DNA was prepared from an overnight culture by using the phenol-chloroform extraction method (4). The quality and concentration of DNA were determined using a Quantus fluorometer with the Quant-iT PicoGreen double-stranded DNA (dsDNA) assay kit (Thermo Fisher Scientific, USA).

DNA samples were sheared into 400- to 500-bp fragments using a Covaris M220 focused acoustic shearer following the manufacturer’s protocol. The Illumina paired-end (PE) libraries were prepared from the sheared fragments using the NEXTflex rapid DNA-Seq kit (Bioo Scientific, USA) and sequenced in 150-bp paired-end mode using the Illumina HiSeq X Ten platform at Majorbio Bio-Pharm Technology, Inc. (Shanghai, China). The sequencing generated 3,449,944 pairs of raw reads totaling 1,041,883,088 bp, giving approximately 362× coverage. The reads were quality trimmed with Trimmomatic v0.36 (5) and assembled using SOAPdenovo v2.04 (https://github.com/aquaskyline/SOAPdenovo2) (6). The resultant assembly totaled 2,874,470 bp with 47 scaffolds, an *N*_50_ value of 232,753 bp, and a GC content of 65.2%.

The genomic scaffolds were analyzed using the I-Sanger Cloud platform from Shanghai Majorbio. Glimmer v3.02 (http://ccb.jhu.edu/software/glimmer/index.shtml) (7) was used for coding DNA sequence (CDS) prediction, tRNA-scan-SE v2.0 (http://trna.ucsc.edu/software/) (8) was used for tRNA prediction, and Barrnap v0.8 (https://github.com/tseemann/barrnap) was used for rRNA prediction. The predicted CDSs were annotated from the nonredundant (NR), Swiss-Prot, Pfam, GO, COG, and KEGG databases using the sequence alignment tools BLAST+ v2.3.0 (9), Diamond v0.8.35 (10), and HMMER v3.1b2 (11). A total of 2,709 CDS genes in addition to 48 tRNAs and 5 rRNAs were annotated for the draft genome sequence, of which 2,653 are protein coding genes. Default parameters were used for all software.

### Data availability.

This whole-genome shotgun sequencing project has been deposited in DDBJ/ENA/GenBank under the accession number JACCDB000000000; the raw reads are available under SRA accession number SRR12179937. This announcement represents the first version of the genome.
